# Seroprevalence of SARS-CoV-2 antibodies among children and adolescents recruited in a malariometric survey in north-eastern Tanzania July 2021

**DOI:** 10.1186/s12879-022-07820-6

**Published:** 2022-11-12

**Authors:** Eric Lyimo, Cyrielle Fougeroux, Anangisye Malabeja, Joyce Mbwana, Paul M. Hayuma, Edwin Liheluka, Louise Turner, Samwel Gesase, Thomas Lavstsen, John P. A. Lusingu, Daniel T. R. Minja, Christian W. Wang

**Affiliations:** 1 National Institute for Medical Research, Tanga Research Centre, P. O. Box 5004, Tanga, Tanzania; 2 Department of Immunology and Microbiology, Centre for Medical Parasitology, University of Copenhagen, Copenhagen, Denmark; 3 AdaptVac Aps, Ole Maaløes Vej 3, 2200 Copenhagen N, Denmark; 4 Department of Infectious Diseases, Copenhagen University Hospital, Copenhagen, Denmark

**Keywords:** COVID-19, Seroprevalence, Spike-protein, Malaria

## Abstract

**Background:**

African countries stand out globally as the region seemingly least affected by the COVID-19 pandemic, caused by the virus SARS-CoV-2. Besides a younger population and potential pre-existing immunity to a SARS-CoV-2-like virus, it has been hypothesized that co-infection or recent history of *Plasmodium falciparum* malaria may be protective of COVID-19 severity and mortality. The number of COVID-19 cases and deaths, however, may be vastly undercounted. Very little is known about the extent to which the Tanzanian population has been exposed to SARS-CoV-2. Here, we investigated the seroprevalence of IgG to SARS-CoV-2 spike protein in two Tanzanian rural communities 1½ years into the pandemic and the association of coinciding malaria infection and exposure.

**Methods:**

During a malariometric survey in July 2021 in two villages in north-eastern Tanzania, blood samples were taken from 501 participants (0–19 years old). Malaria was detected by mRDT and microscopy. Levels of IgG against the spike protein of SARS-CoV-2 were measured by ELISA as well as IgG against five different antigens of *P. falciparum*; CIDRα1.1, CIDRα1.4 and CIDRα1.5 of PfEMP1 and GLURP and MSP3.

**Results:**

The seroprevalence of SARS-CoV-2 IgG was 39.7% (106/267) in Kwamasimba and 32.5% (76/234) in Mkokola. In both villages the odds of being seropositive increased significantly with age (AOR = 1.12, 95% CI 1.07–1.17, *p* < 0.001). *P. falciparum* malaria prevalence by blood smear microscopy was 7.9% in Kwamasimba and 2.1% in Mkokola. 81.3% and 70.5% in Kwamasimba and Mkokola, respectively, showed recognition of minimum one malaria antigen. Residing in Kwamasimba was associated with a broader recognition (AOR = 1.91, 95% CI 1.34–2.71, *p* < 0.001). The recognition of malaria antigens increased significantly with age in both villages (AOR = 1.12; 95% CI 1.08–1.16, *p* < 0.001). Being SARS-CoV-2 seropositive did not associate with the breadth of malaria antigen recognition when adjusting for age (AOR = 0.99; 95% CI 0.83–1.18; *p* = 0.91).

**Conclusion:**

More than a third of the children and adolescents in two rural communities in Tanzania had antibodies to SARS-CoV-2. In particular, the adolescents were seropositive but being seropositive did not associate with the status of coinciding malaria infections or previous exposure. In Tanzania, natural immunity may have developed fast, potentially protecting a substantial part of the population from later variants.

**Supplementary Information:**

The online version contains supplementary material available at 10.1186/s12879-022-07820-6.

## Introduction

The coronavirus, severe acute respiratory syndrome coronavirus 2 (SARS-CoV-2) causing COVID-19, was first reported in Wuhan, China in December 2019 [1, 2]. SARS-CoV-2 belongs to the subfamily of Coronavirinae and genome analyses showed a close relationship with the highly pathogenic strain, SARS-CoV [3, 4]. The positive-sense single-stranded RNA virus is easily transmitted from human to human [4] and has since its discovery spread to all continents leading to the COVID-19 pandemic. The symptoms of the disease range from mild flu-like, including cough and fever, to life-threatening complications. However, even people without symptoms or with a mild course of the disease can transmit the virus [5, 6].

In Africa, when the pandemic occurred, there were major public health concerns due to the existing high prevalence rates for both infectious and non-infectious diseases and the putative adverse consequences of COVID-19 preventive lockdown measures on the prevention and management of other infectious diseases such as malaria [7].

The latest WHO world malaria report revealed an estimated 241 million malaria cases worldwide in 2020 [8]; an increase of 14 million from the previous year [9] resulting in an increase of 12% in malaria deaths to an estimated 627,000. The African region accounted for 96% of all malaria related deaths. Sixty-eight percent (i.e. 47,000) of the estimated additional deaths are believed to be due to service disruptions in the provision of malaria prevention campaigns [8, 10]. Tanzania accounted for 4.1% of all malaria related deaths globally in 2020 [8]. The government of Tanzania decided not to implement a lockdown that would otherwise restrict public access to health services and prevent citizens from working and thereby affecting the ability to afford food and health care, fostering the negative impact of the pandemic [11]. In February 2021, however, the government re-issued guidelines insisting on WHO-recommended measures in the fight against the spread of SARS-CoV2, built local capacity to produce personal protective equipment and on July 24, 2021, the first shipment of COVID-19 vaccines through COVAX was received.

Intriguingly, African countries including Tanzania, stand out globally as the region seemingly least affected by the COVID-19 pandemic as compared to North America and Europe. Besides a younger population and potential pre-existing immunity to a SARS-CoV-2-like virus [12, 13], it has been hypothesized that co-infections or a recent history of *Plasmodium falciparum* malaria infection may be protective of COVID-19 severity and mortality [14, 15]. The number of COVID-19 cases and deaths, however, may be vastly undercounted [16], and a new study reports high seroprevalence across several African countries suggesting higher exposure to SARS-CoV-2 and protection against COVID-19 disease than indicated by surveillance data [17].

Little is known as to what extent the Tanzanian population has been exposed to SARS-CoV-2. So far around 39,000 confirmed cases and 845 deaths due to COVID-19 have been reported to the WHO since the 3rd of January 2020 with the majority (20,607 cases and 553 deaths) reported on the 2nd of August 2021 [18]. Here, as the first to our knowledge, we investigated the seroprevalence of immunoglobulin G (IgG) to SARS-CoV-2 spike protein in a Tanzanian population. The spike protein of the virus binds to host cell surface angiotensin-converting enzyme 2 (ACE2) to initiate entry and cause infection [19] and is the known target of protective immunity [20–22]. IgG targeting SARS-CoV-2 spike protein was measured in plasma from children and adolescents in two rural communities of Kwamasimba (highland village) and Mkokola (lowland village,) in Korogwe District in north-eastern Tanzania. This was done during a malariometric survey in July 2021, just days before the highest recorded number of cases in Tanzania [18].

We further investigated the seroprevalence of spike protein-specific IgG in relation to the prevalence of malaria and the breadth of IgG reactivity to five different antigens of *P. falciparum.* These antigens were three cysteine-rich interdomain region (CIDR) domains (CIDRα1.1, CIDRα1.4 and CIDRα1.5) of *P. falciparum* erythrocyte membrane protein 1 (PfEMP1) associated with the sequestration of infected erythrocytes and severe malaria [23, 24], and two proteins associated with the parasite’s merozoite stage: glutamate-rich protein (GLURP) and merozoite surface protein 3 (MSP3) [25]. Lastly, we investigated if any clinical and demographic data were associated with that of being SARS-CoV-2 seropositive.

## Methods

### Study site and population

A cross-sectional survey was conducted in two rural villages, highland Kwamasimba (KWA) (700 m above sea level) and lowland Mkokola (MKL) (300 m above sea level). The villages are located in Korogwe District, about 100 km from the Indian Ocean coastline and Tanga City in north-eastern Tanzania. According to an updated census survey the population sizes of Kwamasimba and Mkokola were around 2000 each (Malaria Research and Capacity building for field trials in Tanzania (MaReCa) Census survey 2018) during the cross-sectional survey*.*The economic activities are mainly subsistence farming and petty trading. The study communities have been participating in annual surveys since 2003 when the prevalence of *P. falciparum* malaria was higher in Mkokola (78%) than in Kwamasimba (25%) [26] whereas in 2018 the overall point prevalence was 14.3% [27]. The overall malaria prevalence from 2009 to 2021 shows contrasting trends for the two villages (manuscript in preparation). The study population included individuals aged between 0 and 19 years old. Malaria rapid diagnostic test (mRDT) results and haemoglobin levels using HemoCue^®^ (Ångelholm, Sweden) were obtained on site and blood smears for microscopy were prepared at the National Institute for Medical Research, Korogwe Research Laboratory. Study participants who were diagnosed with malaria (mRDT) and with or without anaemia were given appropriate drugs administered as per existing treatment guidelines. Additionally, other diseases such as diarrhoea and skin fungal infections were managed as per the clinician’s discretion.

### Malariometric survey

The cross-sectional survey was conducted from the 19th to the 30th of July 2021 to determine the malaria point prevalence in children and adolescents aged between 0 and 19 years old as previously done [27]. During the survey, participants were asked to provide venous blood samples, which were collected in ethylenediaminetetraacetic acid (EDTA) vacutainers. Participants were randomly selected in stratified age groups (0 to 19) and 529 participants were eligible for enrolment of which 501provided whole blood enough for further analyses. During the survey, a paper-based questionnaire administered by trained clinicians was used to record demographic and clinical data. Clinical symptoms related to malaria in the previous 2 days prior to the survey were recorded and included fever, headache, loss of appetite, vomiting, coughing, and body weakness. Flu was recorded as well to determine whether the symptoms could be ascribed to other causes of febrile illnesses other than malaria. Also, the study participants were asked if they had taken any anti-malaria medication within 14 days prior to the survey date. Throughout the survey, malaria cases were treated per national guidelines based on mRDT results (CareStart™ Malaria Pf/Pv (HRP2/pLDH) Ag Combo RDT (AccessBio, US)). Thick and thin film blood smears were analysed for the presence of *Plasmodium* and for species determination, respectively. The blood slides were prepared using a premade template for quantification of parasite densities and determination of *Plasmodium* species, respectively. The blood slides were fixed with methanol and stained with 5% Giemsa for 20 min and depending on parasite density, parasite count was reported against 200 or 500 white blood cells. Two independent microscopists blinded of the mRDT results, who participate in malaria microscopy proficiency testing, read the blood smears. Plasma samples were obtained by centrifugation of the venous blood and stored at − 80 °C until further testing. During the survey, all national guidelines for the prevention of COVID-19 were followed, including wearing face masks, handwashing, use of hand sanitizers, and distancing whenever possible.

### SARS-CoV-2 spike IgG ELISA

Plasma samples were analysed for SARS-CoV-2 spike IgG titres as described elsewhere [28] with slight modification. Briefly, the 96-well microtiter plates (Nunc MaxiSorp) were coated overnight at 4 °C with 2 µg/mL of recombinant ExpreS2 produced SARS-CoV-2 spike protein in kind contribution from Expres2ion Biotechnologies (amino acid 16-1208, from ABNCoV2 phase I/II study, *manuscript in prep.*) diluted in PBS. Plates were blocked with 3% skimmed milk in PBST (PBS and 0.1% Tween 20) for 1 h at room temperature. Plasma samples were diluted 1:200 with 1% skimmed milk in PBST and 50 µL was added into each well followed by 2 h incubation at room temperature. Microtiterplates were washed three times with PBST, and to detect bound human IgG, rabbit anti-human IgG horseradish peroxidase (HRP) conjugated antibody (Dako, P0214) was then added (1:4000) and incubated at room temperature for 1 h. TMB was added following manufacturer’s instruction and optical densities (ODs) were read at 450 nm. One hundred samples collected from the same two villages in the years 2015 to 2019 were randomly selected and used as negative control and seropositivity was determined as values above three standard deviations of the mean of the 2015 and 2019 samples [29]. To validate the procedure a positive control from WHO (pooled plasma obtained from 11 individuals recovered from SARS-CoV-2 infection) [30], a positive control from a Danish convalescent patient (unknown time of disease) and seven negative pre-COVID-19 Danish donor plasma samples were used in the ELISAs.

### PfEMP1, GLURPR2 and MSP3 IgG ELISA

IgG levels against recombinant domains of three different PfEMP1s (CIDRα1.1, CIDRα1.4 and CIDRα1.5), GLURPR2 and MSP3 [31, 32] were determined in plasma samples as previously described [33]. Sandwich enzyme-linked immunosorbent assay (ELISA) was performed, in brief, for each antigen 5 µg/mL was coated in 96-well microtiterplates (Nunc MaxiSorp) over night at 4 °C. The microplates were blocked with 3% skimmed milk in PBS for 1 h at room temperature. Plasma samples at 1:40 dilution were incubated for 1 h at room temperature and washed three times. To detect bound human IgG, rabbit anti-human IgGHRP conjugated antibody (Dako, P0214) was added (1:3000) and then incubated for 1 h at room temperature followed by three times wash. To detect bound human IgG, TMB (T5525, Sigma-Aldrich) was added following manufacturer’s instruction. Optical densities (ODs) were read at 450 nm on a microtiterplate reader (Multiskan FC, Thermo Scientific). To determine seropositivity the analysis included seven plasma samples from naïve Danish donors as negative controls and the cut-off was set as values above three standard deviations of the mean of the naïve samples. The IgG reactivity to the five antigens was used to generate a score of breadth of malaria antigen recognition of each participant from 0 to 5.

### Data management and analysis

The demographic and clinical data were collected on a paper-based questionnaire and then double-entered into a Microsoft Access database, validated and transferred into Stata v13.0 (Stata Corporation, Texas, USA) for cleaning and merging with ELISA OD values. Statistical analyses were done in STATA and GraphPad Prism v8.4.2 (San Diego, California, USA). In proportional tests, data are presented as percentages, medians, and Chi-square. The Wilcoxon rank-sum (Mann–Whitney) was used to assess the relationship between SARS-CoV-2 spike IgG OD values and malaria prevalence. The correlation between ordered age groups and seropositivity of COVID-19 and malaria was assessed using the Trend test across ordered groups. Multivariate logistic regressions were performed to assess the relationship between SARS-CoV-2 spike seropositivity with malaria prevalence, breadth of malaria antigen recognition, demographic, and clinical data. In the multivariate analysis, a forward selection was done for variables with *p* < 0.2 in the univariate analysis. A *p*-value of < 0.05 was considered statistically significant.

## Results

### Baseline characteristics of the study population by village of residence

In total, 501 study participants were enrolled during the July 2021 cross-sectional malariometric survey; 267 (53.3%) were from the highland village, Kwamasimba and 234 (46.7%) were from the lowland village, Mkokola (Table 1). The survey had a balancing proportion of both sexes (Table 1). The pre-COVID-19 analysis included 100 participants, 46 (46%) from Kwamasimba and 54 (54%) from Mkokola with a similar distribution of age and sex as in the 2021 survey (Additional file 1)*.* In 2021, the *P. falciparum* malaria prevalence by mRDT was 15.4% in Kwamasimba and 6.4% in Mkokola, primarily among children above 5 years of age (Table 1). By blood smear microscopy, the prevalence was 7.9% in Kwamasimba with a median parasitaemia of 2123/µL and 2.1% in Mkokola with a median parasitaemia of 1080/µL. Bed net use was prominent in both villages (> 93%). The study participants in Mkokola had more non-malarial fever (χ^2^ = 12.31, *p* < 0.001) and diarrhoea (χ^2^ = 6.18, *p* < 0.013) than in Kwamasimba, otherwise no marked differences in the clinical characteristics in the two villages was observed; such as haemoglobin levels, coughing, body weakness, headache, body or abdominal pain, loss of appetite, vomiting, and yellowness of eyes (Table 1).

**Table 1:** Baseline characteristics of the study populations July 2021

	VILLAGE		*p-*value
	Kwamasimba	Mkokola	
Age, N = 501, % (n)			
< 1, % (n)	4.9 (13)	6.4 (15)	
1–4, % (n)	15.4 (41)	21.4 (50)	
5–9, % (n)	28.1 (75)	32.5 (76)	
10–14, % (n)	38.6 (103)	26.0 (61)	
15–19, % (n)	13.0 (35)	13.7 (32)	0.044
Sex N = 501			
Male, % (n)	52.1 (139)	46.1 (108)	
Female, % (n)	47.9 (128)	53.8 (126)	0.187
Malaria prevalence by mRDT, N = 501			
Positive, % (n)	15.4 (41)	6.4 (15)	
Negative, % (n)	84.6 (226)	93.6 (219)	0.002
Malaria prevalence by blood smear, N = 501			
Positive, % (n)	7.9 (21)	2.1 (5)	
Negative, % (n)	92.1 (246)	97.9 (229)	0.004
Parasites/µL blood, median, (IQR)	2123 (995–7124)	1080 (985–1320)	
Bed net use, N = 499			
Yes, % (n)	93.3 (249)	99.6 (231)	
No, % (n)	6.7 (18)	0.4 (1)	0.001
Hb, mean (CI 95%)	11.9 (11.7–12.0)	12.4 (12.3–12.7)	

	Kwamasimba n = 245	Mkokola n = 229	
Fever (%) and malaria negative by blood smear	13.1 (32)	25.8 (59)	0.001
Coughing, N = 500			
Yes, % (n)	28.2 (75)	40.6 (95)	
No, % (n)	71.8 (191)	59.4 (139)	0.003
Body weakness, N = 500			
Yes, % (n)	2.6 (7)	5.1 (12)	
No, % (n)	93.6 (249)	86.8 (203)	
NA, % (n)	3.8 (10)	8.1 (19)	0.034
Headache, N = 500			
Yes, % (n)	18.8 (50)	25.6 (60)	
No, % (n)	76.7 (204)	66.7 (156)	
NA, % (n)	4.5 (12)	7.7 (18)	0.039
Body pain, N = 498			
Yes, % (n)	3.7 (10)	4.3 (10)	
No, % (n)	91.7 (243)	86.3 (201)	
NA, % (n)	4.4 (12)	9.4 (22)	0.087
Abdominal pain, N = 499			
Yes, % (n)	16.6 (44)	23.5 (55)	
No, % (n)	78.9 (206)	67.9 (159)	
NA, % (n)	4.5 (12)	8.6 (20)	0.017
Diarrhoea, N = 500			
Yes, % (n)	6.0 (16)	12.4 (29)	
No, % (n)	94.0 (250)	87.6 (205)	0.013
Loss of appetite, N = 500			
Yes, % (n)	3.4 (9)	6.0 (14)	
No, % (n)	93.2 (248)	87.2 (204)	
NA, % (n)	3.4 (9)	6.8 (16)	0.071
Vomiting, N = 500			
Yes, % (n)	2.6 (7)	4.7 (11)	
No, % (n)	97.4 (259)	95.3 (223)	0.215
Yellowness of eyes, N = 499			
Yes, % (n)	0.0 (0)	0.9 (2)	
No, % (n)	100.0 (265)	99.1 (232)	0.132

### Anti-SARS-CoV-2 seroprevalence

**Fig. 1:**
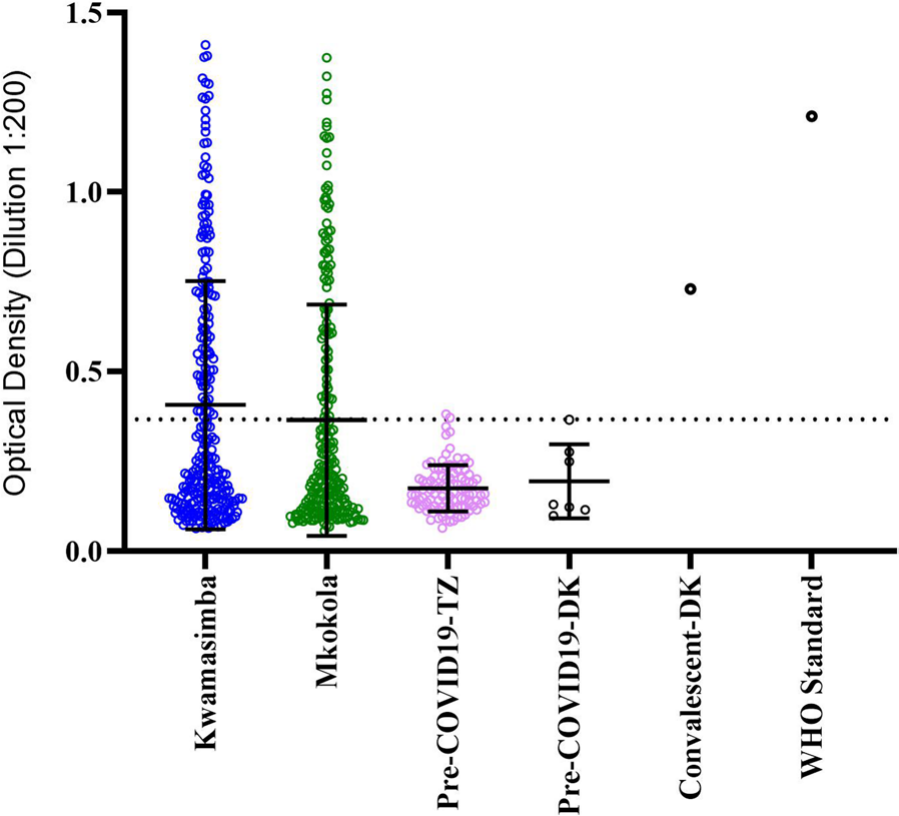
Anti-SARS-CoV-2 IgG levels measured in plasma from 501 individuals by ELISA during a cross-sectional survey in July 2021 in two rural villages Kwamasimba and Mkokola in north-eastern Tanzania, showing mean and standard deviation. The cut-off is represented by a dotted line. Plasma samples from before the COVID-19 pandemic were used as negative controls; a Danish convalescent sample and the WHO Standard [30] were used as positive control

The IgG recognition of theSARS-CoV-2 spike protein of the 501 study participants and positive and negative controls are shown in Fig. 1. The difference in seroprevalence between the villages, 39.7% in Kwamasimba and 32.5%in Mkokola, was not statistically significant (χ^2^ = 2.8, *p* = 0.094), however, the seropositivity increased with age in both villages from around 20% in the 1–4-year-olds (17.1% and 22.0% in Kwamasimba and Mkokola, respectively) to at least 50% in the older age group of 15–19 years; 68.6% and 50% in Kwamasimba (z-score = 4.75, *p* = 0.001) and Mkokola (z-score = 2.67, *p* = 0.007), respectively (Table 2). Among the < 1-year olds the seroprevalence was 38.5% and 33.3%) in Kwamasimba and Mkokola, respectively (Table 2). When combining the two villages, among the 0–5 months old: 55.6% (5/9) were seropositive and among the 6–11 months old: 26.3% (5/19) were seropositive but the difference was not statistically significant (Additional file 2).

**Table 2:** SARS-CoV-2 seropositivity, malaria prevalence and recognition of malaria antigens in Kwamasimba and Mkokola

Age groups	N	Kwamasimba					Mkokola				
		n	SARS-CoV-2 seropositivity	Malaria antigen recognition	Malaria (BS)	Malaria (mRDT)	n	SARS-CoV-2 seropositivity	Malaria antigen recognition	Malaria (BS)	Malaria (mRDT)
< 1	28	13	38.5% (5)	46.2% (6)	0.0%(0)	0.0% (0)	15	33.3% (5)	40.0% (6)	0.0% (0)	6.7% (1)
1–4	91	41	17.1% (7)	65.9% (27)	0.0% (0)	7.3% (3)	50	22.0% (11)	34.0% (17)	4.0% (2)	2.0% (1)
5–9	151	75	25.3% (19)	78.7% (59)	6.7% (5)	13.3% (10)	76	25.0% (19)	81.6% (62)	1.3% (1)	6.6% (5)
10–14	164	103	49.5% (51)	89.3% (92)	10.7% (11)	21.4% (22)	61	41.0% (25)	85.3% (52)	1.6% (1)	8.2% (5)
15–19	67	35	68.6% (24)	94.3% (33)	14.3% (5)	17.1% (6)	32	50.0% (16)	87.5% (28)	3.1% (1)	9.4% (3)
Total	501	267	39.7% (106)	81.3% (217)	7.9% (21)	15.4% (41)	234	32.5% (76)	70.5% (165)	2.1% (5)	6.4% (15)
*p-*value			0.001	0.001	0.006	0.017		0.007	0.001	0.988	0.221
z-score			4.75	4.90	2.74	2.38		2.67	6.07	0.02	1.22

### Serorecognition of *Plasmodium falciparum* malaria antigens

**Fig. 2:**
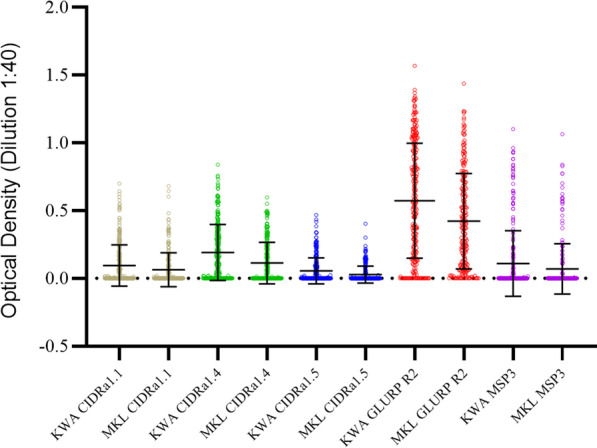
501 individual anti-malarial antigen IgG levels measured in plasma by ELISA during a cross-sectional survey in July 2021 in two rural villages Kwamasimba (KWA) and Mkokola (MKL) in north-eastern Tanzania, showing mean and standard deviation. Naïve Danish plasma samples were used as control

The IgG reactivity in the 501 plasma samples to five *P. falciparum* derived antigens were evaluated by ELISA. Most study participants, 81.3% and 70.5% (χ^2^ = 7.973, *p* = 0.005) in Kwamasimba and Mkokola, respectively, showed recognition of minimum one antigen (Fig. 2 and Table 2). The serorecognition of GLURP R2 (74.9% and 67.9% in Kwamasimba and Mkokola, respectively; χ^2^ = 2.972, *p* = 0.09) and MSP3 (14.2% and 9.0% in Kwamasimba and Mkokola, respectively; χ^2^ = 3.318, *p* = 0.07) was similar between the two villages. On the contrary, the serorecognition of the three PfEMP1 domains was significantly higher in Kwamasimba than in Mkokola: CIDRα1.1: 24.7% and 15.8% (χ^2^ = 6.058, *p* = 0.01); CIDRα1.4: 38.6% and 24.4% (χ^2^ = 11.597, *p* = 0.001); CIDRα1.5: 3.8% and 0.8% (χ^2^ = 4.457, *p* = 0.04), respectively. The number of malaria antigens recognized was significantly increased with age in both villages (Trend test across ordered groups, Kwamasimba, z-score = 4.90, *p* < 0.001, and Mkokola, z-score = 6.07, *p* < 0.001) (Table 2).

### Association of SARS-CoV-2 seroprevalence and malaria

The association of SARS-CoV-2 seropositivity, malaria prevalence and breadth of malaria antigen recognition was explored by logistic regression (Table 3). There was no association between SARS-CoV-2 seropositivity and malaria prevalence, neither by mRDT nor blood smear microscopy (Table 3). Being SARS-CoV-2 seropositive was, however, associated with the breadth of malaria antigen recognition in the univariate regression analysis (OR = 1.17; 95% 1.01–1.36; *p* = 0.04) but in the multivariate regression analysis the association disappeared (AOR = 0.99; 95% CI 0.83–1.18; *p* = 0.91) (Table 3).

**Table 3:** Association of SARS-CoV-2 seroprevalence with malaria prevalence, breadth of malaria antigen recognition and age

Characteristic	N	Crude OR (95% CI)	p-value	Adjusted OR (95% CI)	p-value	Overall *p*-value
Breadth of malaria antigen recognition						0.001
Antigen recognition	501	1.17 (1.01–1.36)	**0.04**	0.99 (0.83–1.18)	0.91	
Malaria prevalence by mRDT						
Neg	445	Ref	Ref	Ref		
Pos	56	1.48 (0.84–2.59)	0.17	1.21 (0.64–2.28)	0.56	
Age of participants						
Age in years	501	1.12 (1.08–1.17)	**0.001**	1.12 (1.07–1.17)	**0.001**	

Bold indicates* p*-values < 0.05

### Association of SARS-CoV-2 seroprevalence and demographic and clinical data

The association of SARS-CoV-2 seropositivity and demographic and clinical characteristics was also explored by logistic regression (Table 4). The SARS-CoV-2 seropositivity did not associate with any of the investigated demographic and clinical data, with the exception of age where the risk of being seropositive increased significantly with age (AOR = 1.13, 95% CI 1.08–1.19, *p* < 0.001).

**Table 4:** The association of SARS-CoV-2 seroprevalence and demographic and clinical data

Characteristic	N	Crude OR (95% CI)	p-value	Overall p-value	Adjusted OR (95% CI)	p-value	Overall *p*-value
Age of participants							**0.001**
Age in years	501	1.12 (1.08–1.17)	**0.001**		1.13 (1.08–1.19)	**0.001**	
Village of residence							
Mkokola	234	Ref	Ref		Ref		
Kwamasimba	267	1.37 (0.95–1.98)	0.09		1.24 (0.83–1.86)	0.28	
Haemoglobin levels							
Hb	501	1.13 (1.00–1.27)	0.05		0.99 (0.86–1.13)	0.86	
Cough							
Yes	170	Ref	Ref		Ref		
No	330	1.36 (0.92–2.01)	0.12		1.19 (0.78–1.82)	0.41	
Body pain							
Yes	20	Ref	Ref	Ref	Ref		
No	444	0.46 (0.19–1.14)	0.09	0.16	0.73 (0.28–1.86)	0.51	
NA	34	0.34 (0.10–1.08)	0.07		1.69 (0.46–6.15)	0.43	

Bold indicates* p*-values < 0.05

### Association between breadth of malaria antigen recognition and demographic and clinical data

The association between the breadth of malaria antigen recognition and demographic and clinical data was explored by ordered logistic regression (Table 5). Residing in Kwamasimba was associated with a broader recognition of malaria antigens (AOR = 1.91 95% CI 1.34–2.71, *p* < 0.001) and the recognition increased significantly with age in both villages AOR = 1.12; 95% CI 1.08–1.16, *p* < 0.001). The breadth of malaria antigen recognition was inversely associated with a previous 2-day clinical history of having fever (AOR = 0.41; 95% CI 0.25–0.67, *p* < 0.001) and associated with not having body pain (AOR = 2.83; 95% CI 1.03–7.75, *p* < 0.04).

**Table 5:** The association of breadth of malaria antigen recognition and demographic and clinical data

Characteristic	N	Crude OR (95% CI)	p-value	Overall p-value	Adjusted OR (95% CI)	p-value	Overall p-value
Age of participants							**0.001**
Age in years	501	1.16 (1.12–1.20)	**0.001**		1.12 (1.08–1.16)	**0.001**	
Village of residence							
Mkokola	234	Ref	Ref		Ref		
Kwamasimba	267	1.88 (1.35–2.60)	**0.001**		1.91 (1.34–2.71)	**0.001**	
Bed net use							
Yes	480	Ref	Ref		Ref		
No	19	3.95 (1.64–9.51)	**0.002**		2.27 (0.87–5.88)	0.09	
Fever							
Yes	111	Ref	Ref		Ref		
No	389	0.45 (0.31–0.68)	**0.001**		0.41 (0.25–0.67)	**0.001**	
Cough							
Yes	170	Ref	Ref		Ref		
No	330	1.26 (0.90)	0.18		1.20 (0.83–1.74)	0.32	
Body weakness							
Yes	19	Ref	Ref	Ref	Ref		
No	452	0.39 (0.17–0.89)	**0.03**	**0.001**	1.00 (0.36–2.80)	1.00	
NA	29	0.06 (0.02–017)	**0.001**		1.27 (0.0–18.54)	0.86	
Headache							
Yes	110	Ref	Ref	Ref	Ref		
No	360	0.40 (0.27–0.59)	**0.001**	**0.001**	0.65 (0.40–1.07)	0.09	
NA	30	0.05 (0.02–0.12)	**0.001**		0.06 (0.01–0.46)	**0.01**	
Body pain							
Yes	20	Ref	Ref	Ref	Ref		
No	444	0.60 (0.60–0.26)	0.21		2.83 (1.03–7.75)	**0.04**	
NA	34	0.10 (0.03–0.27)	**0.001**		4.35 (0.50–37.61)	0.18	
Abdominal pain							
Yes	99	Ref	Ref	Ref	Ref		
No	368	0.69 (0.46–1.04)	0.08		0.85 (0.55–1.33)	0.48	
NA	32	0.10 (0.05–0.23)	**0.001**		1.63 (0.25–10.78)	0.61	
Loss of appetite							
Yes	23	Ref	Ref	Ref	Ref		
No	452	0.37 (0.17–0.80)	**0.01**		0.67 (0.28–1.56)	0.35	
NA	25	0.05 (0.02–0.16)	**0.001**		0.53 (0.06–4.93)	0.58	

Bold indicates* p*-values < 0.05

## Discussion

Serum antibody testing has the potential to detect previous COVID-19 infections. We therefore measured the seroprevalence of SARS-CoV-2 spike protein IgG in blood samples from two rural villages in Tanzania during the pandemic, July 2021. Around a third of the participants were positive for antibodies recognizing the spike protein and the seroprevalence increased with age, from ~ 20% in the 1–4-year-olds to ~ 60% in the 15–19-year-olds. Global seroprevalence has been estimated to be ~ 60% by September 2021 from either infection or vaccination, and children under 10 years and adults above 60 were less likely to be seropositive than 20–29-year-old [34]. In Africa, overall SARS-CoV-2 seroprevalence seemed to rise from 3% in April–June 2020 to 65% in July–September 2021 [17], up to ~ 87% by December 2021, mainly due to infection [34], whereas in high-income countries in Europe seroprevalence rose to ~ 96% due to infection and vaccination [34].

The antibody titers were at similar levels as the tested convalescent samples used. COVID-19 in children and adolescents is associated with asymptomatic or mild illness and much lower mortality than in adults [35]. Whether or not the seropositive participants had symptoms at the time of infection, diagnosis was not available to establish actual infection of SARS-CoV-2 and the different levels of antibody titer may represent different time from infection to antibody measurement. Furthermore, we did not test the neutralizing potential of the detected antibodies.

In countries in North America and Europe public health measures, such as school closures, have minimized children’s exposure to SARS-CoV-2 but seroprevalence of SARS-CoV-2 antibodies seem similar irrespective of age [36, 37]. Interestingly, in our study, the adolescents showed markedly higher seroprevalence than the children, which could be due to a more social and risk-taking behaviour of adolescents [38]. It is well known that maternal IgG can be obtained by the fetus protecting the baby after birth [39]. The titre of SARS-CoV-2 IgG in infants has been shown to correlate to that of their mothers [40]. This may explain why infants in our study showed higher recognition of SARS-CoV-2 than the children 1–4 and 5–9-year-old age groups, in particular among the infants under 6 months of age.

The seroprevalence of SARS-CoV-2 varied markedly among geographic regions early in the pandemic and was higher in high-income countries with very high human development index levels [36], however, uneven access to health care and diagnostics, and self-medicating practices outside the health system may also be reasons for the geographic variation [16, 34, 41]. Recently, the exposure to SARS-CoV-2 has also been associated with disadvantageous living conditions and overcrowded households [42]. In a (preprint) meta-analysis of the seroprevalence in Africa, the highest seroprevalences were found in adults (vs. children) and in urban areas (vs. rural) [17] with Africa being the world’s second-most populous continents, but also one of the least urbanized [43]. The observed seroprevalences in our study are below of what has been reported in other studies on the African continent at that time [17], however, we investigated only children and adolescents in rural areas. In Tanzania, the government did not implement a lockdown and with the limited testing facility available little is known about the overall exposure of SARS-CoV-2 and one can only guess at what the exposure has been in highly densely populated cities, such as Dar es Salaam. Archived blood samples, if available, may shed light on the epidemiology of SARS-CoV-2 during the pandemic in Tanzania.

Previous exposure to SARS-CoV-2-like virus inducing cross-reactive antibodies have been shown using ELISA in both Sierra Leone (52% cross-reactivity) and the Democratic Republic of Congo (19.2% cross-reactivity) [12, 13], respectively. In our study, IgG from archived plasma samples collected in the same two villages of our study, before the pandemic, only two out of 100 individuals reacted with the SARS-CoV-2 spike protein used here (wild-type sequence [28]; Expres2ion Biotechnologies), encompassing both the S1 and S2 subunits [44] with the used cut-off. This lack of reactivity indicates that cross-reactivity in our setting may be due to our plasma samples being taken from children and adolescents or because different recombinant proteins were used. The study in Sierra Leone used a recombinant N-protein [12], whereas in Congo [13] an N-protein, S1 protein, the receptor binding domain (RBD) of the S1 protein, the N-terminal domain of the S1 protein, and the S2 protein was used, only showing no cross-reactivity to the RBD. However, as the individuals, defined as being either SARS-CoV-2 seropositive or negative in our study, actual COVID-19 infections were not confirmed by PCR; hence we may risk mislabelling certain individuals with the used cut-off, potentially underestimating the cross-reactivity.

Medical conditions such as cancer, chronic lung diseases, heart conditions, diabetes, but as well as infectious diseases such as tuberculosis [45] and HIV [46] have been associated with higher risk of severe illness from COVID-19. In our study, we had the opportunity to investigate the individual seroprevalence with that of a coinciding malaria infection and previous exposure to *P. falciparum*. It has been hypothesized that co-infections or a recent history of malaria infection could be protective of COVID-19 severity and mortality [14, 15]. However, we did not find any association, negative or positive, when adjusting for the age-related increased exposure to both pathogens.

This current study was limited by an overall malaria prevalence of only 5.2% by blood smear microscopy (11.2% by mRDT) and by not investigating coinciding SARS-CoV-2 infections using PCR or IgM, or severe illness from COVID-19 infection. Furthermore, the used questionnaire was not designed to capture clinical information linked to a potential SARS-CoV-2 infection since this study was done on already collected samples from a malariometric cross-sectional survey. A longitudinal cohort study would have been more appropriate to investigate an association of the history of malaria exposure and the incidence of COVID-19 infections.

We did, however, find a higher serorecognition of malaria antigens in Kwamasimba, in particular among the 1–4-year-olds, which indicates a higher prior exposure to malaria parasites [31] as reflected in the prevalence and parasitaemia, both being more than twice as high in Kwamasimba than in Mkokola. A broader recognition of antigens associated with fewer cases of fever, but also with lower levels of haemoglobin which could indicate higher protection from apparent symptoms but not from increased risk of anaemia [27].

## Conclusion

We found that more than a third of children and adolescents in rural Tanzania were having antibodies to SARS-CoV-2. This was almost one and a half year into the pandemic, few days prior to the highest number of recorded cases in the country, prior COVID-19 vaccination, and prior to the rapid spreading of the omicron variant (B.1.1.529) [47]. In a country that did not implement lockdown, natural immunity may have developed fast, potentially protecting a substantial part of the population from later variants such as the omicron [48].

## Supplementary Information

**Additional file 1. **Baseline characteristics of the study populations from before the COVID-19 pandemic.

**Additional file 2** The number of SARS-CoV-2 seropositive and negative infants in Kwamasimba and Mkokola villages based on age in months.

## Data Availability

The datasets used and analysed in this study are available from the grant holder (JPAL) through the corresponding author (EL) on reasonable request.

## Abbreviations

ACE2: Angiotensin-converting enzyme 2
AOR: Adjusted odds ratio
CIDR: Cysteine-rich interdomain region
COVAX: COVID-19 Vaccines Global Access
COVID-19: Coronavirus disease 2019
EDTA: Ethylenediaminetetraacetic acid
ELISA: Enzyme-linked immunosorbent assay
GLURP R2: Glutamate rich protein-region 2
HRP: Horseradish peroxidase
IgG: Immunoglobulin G
KWA: Kwamasimba village
MKL: Mkokola village
mRDT: Malaria rapid diagnostic test
MSP3: Merozoite surface protein 3
*P. falciparum*: *Plasmodium falciparum*
PBST: Phosphate-Buffered Saline Tween20
PfEMP1: *Plasmodium falciparum* Erythrocyte membrane protein 1
SARS-CoV-2: Severe Acute Respiratory Syndrome Coronavirus 2
TMB: 3,3′5,5′-Tetramethylbenzidine
