# Supplementary material for: Seroprevalence of SARS-CoV-2 antibodies among children and adolescents recruited in a malariometric survey in north-eastern Tanzania July 2021
Source: BMC Infect Dis. 2022 Nov 12;22:846. doi: 10.1186/s12879-022-07820-6 (PMC9652923; doi:10.1186/s12879-022-07820-6)
Supplement: Supplementary file 1 — Additional file 1. Baseline characteristics of the study populations from before the COVID-19 pandemic. [file 12879_2022_7820_MOESM1_ESM.docx]

**Additional file 1** Baseline characteristics of the study populations from before the COVID-19 pandemic

|  | VILLAGE | |
| --- | --- | --- |
| Age, N=100, % (n) | Kwamasimba  N = 46 | Mkokola  N = 54 |
| <1, % (n) | 4.3 (2) | 1.9 (1) |
| 1 – 4, % (n) | 32.6 (15) | 27.8 (15) |
| 5 – 9, % (n) | 19.6 (9) | 29.7 (16) |
| 10 – 14, % (n) | 34.8 (16) | 33.3 (18) |
| 15 – 19, % (n) | 8.7 (4) | 7.4 (4) |
|  |  |  |
| Sex N=100 |  |  |
| Male, % (n) | 43.5 (20) | 40.7 (22) |
| Female, % (n) | 56.5 (26) | 59.3 (32) |
|  |  |  |
| Malaria prevalence by mRDT, N=100 |  |  |
| Positive, % (n) | 10.9 (5) | 20.4 (11) |
| Negative, % (n) | 89.1 (41) | 79.6 (43) |
|  |  |  |
| Malaria prevalence by Blood Smear, N=100 |  |  |
| Positive, % (n) | 2.1 (1) | 11.1 (6) |
| Negative, % (n) | 97.9 (45) | 88.9 (48) |
|  |  |  |
| Parasites/µl blood, median, (IQR) | 880 (880 – 880) | 16000 (2240 – 57040) |
|  |  |  |
| Bed net use, N=100 |  |  |
| Yes, % (n) | 67.4 (31) | 94.4 (51) |
| No, % (n) | 32.6 (15) | 5.6 (3) |
|  |  |  |
| Hb, mean (CI 95%) | 12.3  (11.7–12.9) | 12.3  (11.9–12.7) |
|  |  |  |
|  | Kwamasimba n=45 | Mkokola n=48 |
| Fever (%) and malaria negative by blood smear | 8.9 (4) | 14.6 (7) |
|  |  |  |
| Coughing, N=100 |  |  |
| Yes, % (n) | 43.5 (20) | 31.5 (17) |
| No, % (n) | 56.5 (26) | 68.5 (37) |
|  |  |  |
| Body Weakness, N=100 |  |  |
| Yes, % (n) | 2.2 (1) | 1.9 (1) |
| No, % (n) | 82.6 (38) | 94.4 (51) |
| NA, % (n) | 15.2 (7) | 3.7 (2) |
|  |  |  |
| Headache, N=100 |  |  |
| Yes, % (n) | 8.7 (4) | 20.6 (11) |
| No, % (n) | 71.7 (33) | 75.9 (41) |
| NA, % (n) | 19.6 (9) | 3.7 (2) |
|  |  |  |
| Body Pain, N=100 |  |  |
| Yes, % (n) | 0.0 (0) | 5.6 (3) |
| No, % (n) | 80.4 (37) | 90.7 (49) |
| NA, % (n) | 19.6 (9) | 3.7 (2) |
|  |  |  |
| Abdominal Pain, N=100 |  |  |
| Yes, % (n) | 15.2 (7) | 25.9 (14) |
| No, % (n) | 69.5 (32) | 70.4 (38) |
| NA, % (n) | 15.2 (7) | 3.7 (2) |
|  |  |  |
| Diarrhoea, N=100 |  |  |
| Yes, % (n) | 4.3 (2) | 1.8 (1) |
| No, % (n) | 95.7 (44) | 98.2 (53) |
|  |  |  |
| Loss of appetite, N=100 |  |  |
| Yes, % (n) | 2.2 (1) | 5.6 (3) |
| No, % (n) | 82.6 (38) | 90.7 (49) |
| NA, % (n) | 15.2 (7) | 3.7 (2) |
|  |  |  |
| Vomiting, N=100 |  |  |
| Yes, % (n) | 2.2 (1) | 7.4 (4) |
| No, % (n) | 97.8 (45) | 92.6 (50) |
|  |  |  |
| Yellowness of eyes, N=100 |  |  |
| Yes, % (n) | 0.0 (0) | 1.9 (1) |
| No, % (n) | 100.0 (46) | 98.1 (53) |
