# Supplementary material for: Seroprevalence of SARS-CoV-2 antibodies among children and adolescents recruited in a malariometric survey in north-eastern Tanzania July 2021
Source: BMC Infect Dis. 2022 Nov 12;22:846. doi: 10.1186/s12879-022-07820-6 (PMC9652923; doi:10.1186/s12879-022-07820-6)
Supplement: Supplementary file 2 — Additional file 2 The number of SARS-CoV-2 seropositive and negative infants in Kwamasimba and Mkokola villages based on age in months. [file 12879_2022_7820_MOESM2_ESM.docx]

**Additional file 2** The number of SARS-CoV-2 seropositive and negative infants in Kwamasimba and Mkokola villages based on age in months.

| Age in Months | Kwamasimba and Mkokola, N=28 | |
| --- | --- | --- |
|  | Seropositive | Seronegative |
| 1 | 1 | 1 |
| 2 | 0 | 1 |
| 3 | 0 | 0 |
| 4 | 2 | 1 |
| 5 | 2 | 1 |
| 6 | 1 | 3 |
| 7 | 0 | 0 |
| 8 | 0 | 5 |
| 9 | 2 | 5 |
| 10 | 2 | 1 |
| 11 | 0 | 0 |
| Total | 10 | 18 |
